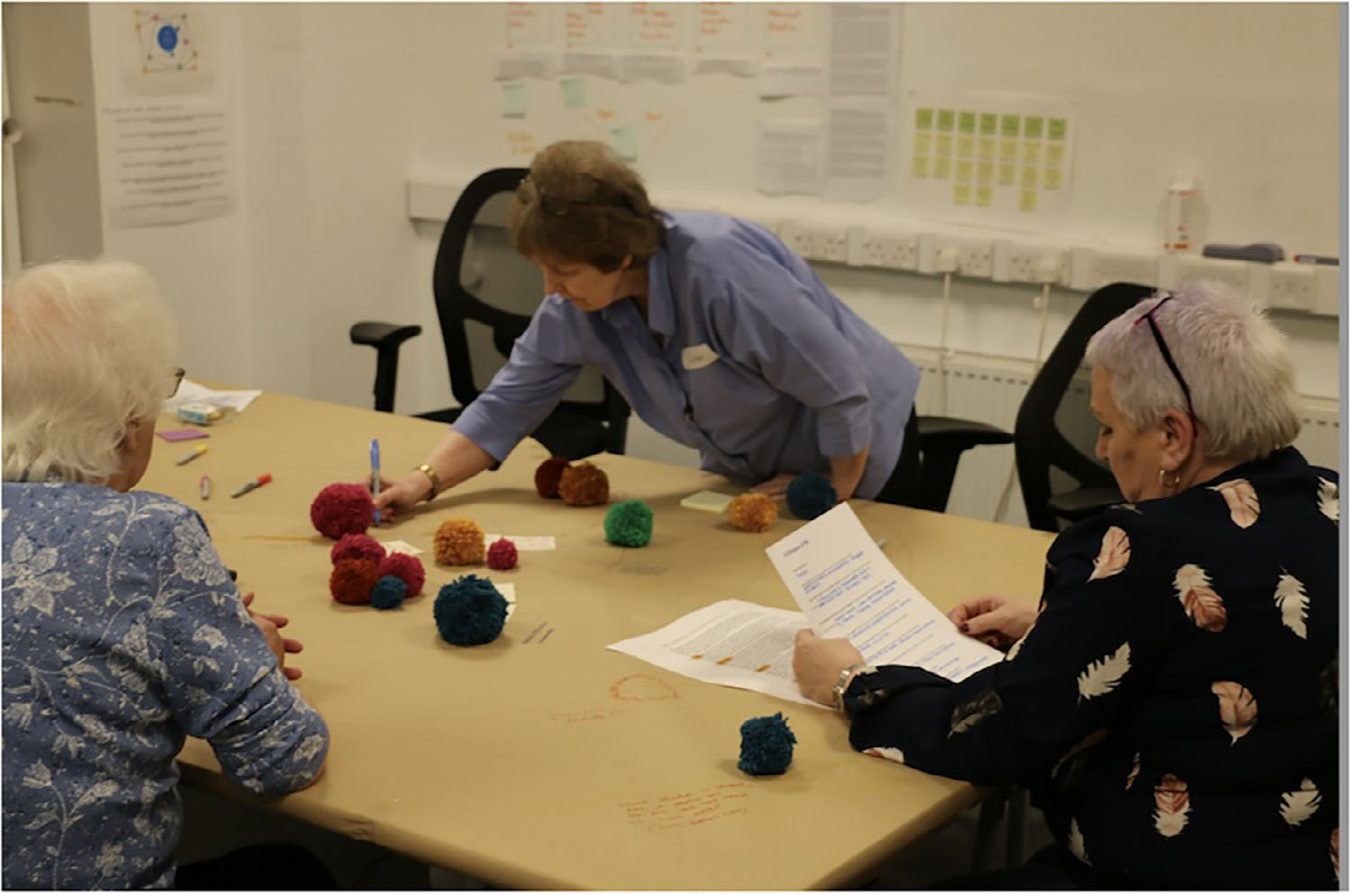# Interactive workshops exploring caregivers’ perceptions of engagement among people with advanced dementia

**DOI:** 10.1002/alz70858_104835

**Published:** 2025-12-26

**Authors:** Kandianos Emmanouil Sakalidis, Henry Collingham, Arlene Astell

**Affiliations:** ^1^ Northumbria University, Newcastle upon Tyne, United Kingdom

## Abstract

**Background:**

Engagement can encourage meaningful connections and reduce the risk of social isolation for people living with dementia (1). However, how to engage people living with advanced dementia is understudied, in part due to the complexity of understanding what engagement is and how to measure it (2). This study aimed to: (i) explore caregivers’ perceptions of engagement, (ii) identify existing measurement practices, and (iii) determine factors that promote engagement among people living with advanced dementia.

**Method:**

Nineteen family and formal caregivers (74% women) were divided into groups and participated in two rounds of interactive workshops. The eight workshops in total (Mean duration = 280 minutes) included co‐creating vignettes, team‐based activities, guided discussions, and video observations relevant to engagement in advanced dementia. The workshops were video‐recorded with content analysis used to analyze the workshop transcripts.

**Result:**

Content analysis revealed key themes relevant to the three research aims. (i) Regarding caregivers’ perceptions, engagement was recognized as a ‘*collaborative concept’* between the person and their social environment, observed through ‘*verbal and non‐verbal cues’* (e.g., eye gaze) and influenced by ‘*external factors’* (e.g., music). (ii) For the identification of existing measurement practices, caregivers highlighted the ‘*lack of standardized tools and formal policies’*, and the reliance on ‘*personalized informal approaches and records*’ to measure and report engagement. (iii) In terms of determining factors that promote engagement, the importance of considering ‘*contextual influences’* (e.g., type of activity) and ‘*personalized engagement strategies’* based on the person's interests were highlighted. Additionally, understanding the ‘*dynamics of interaction with the social environment’* as well as the ‘communication and interaction’ patterns of those involved were stressed.

**Conclusion:**

This study revealed that caregivers perceive engagement as a behavioral response to environmental cues. The findings indicate that both family and formal caregivers believe that engagement can be assessed and that environmental factors exert an important influence. However, the lack of formal tools and procedures makes assessing engagement a challenging process in care settings. This study highlights the need for a rigorous approach to evaluating engagement in people living with advanced dementia.